# The temperature effect on perceived income

**DOI:** 10.1038/s41598-024-56852-2

**Published:** 2024-03-14

**Authors:** Ang Sun, Wang Xiang, Xu Jiang

**Affiliations:** School of Finance, Renmin University of China, Beijing, China

**Keywords:** Temperature, Income, Survey methodology, Mood, Cognitive capacity, Human behaviour, Environmental economics, Psychology and behaviour

## Abstract

Extensive research has focused on the impact of weather on working capacity and income. However, in regions where income data largely relies on surveys, a pivotal yet underexplored question is whether weather not only influence real income but also introduce biases into survey-collected income data. We analyze longitudinal data from the China Health and Nutrition Survey and corresponding weather records from the Global Surface Summary of the Day, and uncover a negative correlation between survey-day temperature and self-reported annual income from the previous year. With a series of robustness checks, we confirm that the effect is primarily driven by behavioral factors rather than actual income changes. And threshold regression analyses show that the impact of temperature is more pronounced on hot days and relatively subdued or even reversed on cooler days. Further analyses indicate that mood, rather than cognitive capacity, plays a central role in causing the observed downward bias.

## Introduction

Increasing occurrences of adverse weather events have seized the attention of both scholars and policymakers, compelling a thorough exploration of the influence of weather on working ability and income—a relationship intrinsically tied to economic development and disparities, both on a national and international scale^1–4^. However, in developing world where the payroll system is not well established, the main data source for income is surveys. Given that weather exerts a notable impact on the reliability of subjective responses in surveys, particularly those related to life satisfaction^5–7^ and happiness^8,9^, an underexplored yet pivotal question emerges: Could weather conditions not only influence real income but also introduce biases into income data collected through surveys?

In this study, we aim to explore how weather conditions affect individual’s self-reporting behavior. In particular, we focus on whether temperature on the survey date could systematically bias self-reported income from the previous year, because temperature is easy to measure and the heat stress is a universal physiological mechanism^10^. We fit an individual-level fixed-effects model using longitudinal individual data from the China Health and Nutrition Survey (CHNS) combined with weather data extracted from the Global Surface Summary of the Day (GSOD). We find that in instances of elevated temperatures (e.g., one standard deviation above the mean) compared to colder conditions (e.g., one standard deviation below the mean), respondents exhibit an average reduction of 581 RMB (approximately 80 USD) in their reported annual income. This decrease corresponds to about 5 percent of the mean income. This magnitude is not negligible given that the increase in annual income is only 11.8 percent according to the National Bureau of Statistics of China (NBSC). And the impact of temperature is more pronounced on hot days (e.g., survey days with average temperature greater than 77 °F).

We conduct a series of analyses showing that this decrease in reported annual income from the previous year is unlikely to be attributed to the reduction in real income/production. In particular, we address the concern that high temperature on the survey date could reflect the trend of climate change, such as global warming, which compromises the productivity of labor and/or land. First, we adopt the difference between the weather at the date of interview and the corresponding weekly average as our weather metrics to isolate the effect of transient weather conditions, which is arguably regarded as a natural experiment. Second, we replace the temperature on the survey date with the temperature one day before or after the survey. These temperatures have no effect on self-reported incomes. Third, we find that the temperature on the survey date only affects self-reported income but has no statistically significant effect on imputed income, which is predicted based on the average incomes from previous and subsequent waves and on community-level information.

We further investigate the mechanisms through which temperature affects self-reported income. Prior research has established that weather can affect both people’s mood and cognitive capacity. Both are highly likely to affect how a survey respondent could retrieve information in the past. In this study, we aim to differentiate the two mechanisms with a series of heterogeneity analyses. Regarding the link between weather and mood, we find that high temperature significantly decreases respondents’ reports of life satisfaction and makes them feel more upset, uncontrolled, burdened and overwhelmed, aligning with existing research findings. Bower^11^, Watkins et al.^12^ and Drace^13^ suggest that a negative mood will lead individuals to retrieve information with the same emotional content, which is referred to as the mood congruence effect. Therefore, being in a depressed mood increases the tendency to remember negative events, such as income loss. In addition, as documented by^14–17^, and numerous other researchers, women and less healthy individuals are more emotionally sensitive to weather. In line with these findings, our study reveals that the impact of temperature on self-reported income is more pronounced within subgroups that exhibit greater susceptibility to mood fluctuations triggered by temperature changes.

Alternatively, we explore whether self-reporting biases can be linked to a broader influence of temperature on the cognitive capacity of survey respondents. Following the approach of Jeong et al.^18^, we construct a measure of questionnaire completeness for health and daily habit related questions within the CHNS dataset. Our analysis indicates that temperature does not significantly impact respondents’ ability to complete these questions, whether for the entire population or specific subgroups such as women, the less healthy, or rural residents. As a sensitivity check to assess the reliability of questionnaire completeness as an indicator of cognitive capacity, we construct a similar measure in the China Family Panel Studies (CFPS) dataset, and reveal a significant correlation between questionnaire completeness and respondents’ performance across various cognitive tests. Our findings thus suggest that cognitive capacity is unlikely to be a key driver of the observed underreporting of income during periods of high temperature.

This paper fits into the broad field of weather effects on human behavior. Although some of these effects are unremarkable, such as outdoor exercise (for a systematic review, see^19^), diverse activities are found to be unexpectedly yet dramatically influenced by weather conditions. Examples include but are not limited to academic performance, suicide, and crime^20^, helping behavior^21^, investment^22,23^, saving^24^, consumption^25,26^, and even court decisions^27^. The current study contributes to several areas of this literature.

First, this study addresses the issue of weather-related survey bias, which has received growing attention from survey methodologists and economists in recent years. Specifically, Connolly^5^ explored the weather effect on self-reported well-being in the U.S. and concluded that survey respondents preferred mild weather with less rain. Subsequent studies continued to explore this impact, but the evidence is mixed. On the one hand, Lucas et al.^28^ and Schmiedeberg and Schröder^29^ reported no consistent evidence supporting the existence of such an effect. On the other hand, when a panel data framework and more accurate weather records were adopted, Feddersen et al.^6^ and Barrington-Leigh and Behzadnejad^7^ suggested that weather did indeed influence self-reported subjective measures of well-being, such as life satisfaction and happiness. In contrast to previous studies that focused on subjective responses, our research delves into self-reported objective measures within surveys. Expanding upon this literature, we employ an individual fixed effect panel data analysis to estimate the weather impact on individual self-reported income.

Second, this paper provides supplementary evidence that daily variation in weather may affect an individual’s mood. While the effects of seasons on mood have been extensively studied (e.g.,^30^), the relationship between daily weather and mood has produced mixed results. Some studies have reported that people prefer warm weather^21^, low humidity^31^, and high sunshine^32^, while others have found no significant relationships between mood and any weather variable^33^. Keller et al.^8^ revealed that warm and sunny weather can improve cognitive flexibility and positive mood, but the effects are contingent upon various factors, including the type of weather and the amount of time spent outdoors. Denissen et al.^9^ demonstrated that rainy and windy weather was associated with lower mood, while temperature and sunshine have more complex effects. Baylis et al.^34^ found that hot weather worsens expressed sentiment. Additionally, these impacts were found to be idiosyncratic across individuals with different personality traits^9,35^. In the extensive literature on this topic, Hua et al.^16^ reported findings that are most similar to ours. Through a longitudinal survey conducted in China, these authors noted a significant rise in depression rates during high temperatures, especially among specific demographics such as elderly individuals, women, and those engaged in the agricultural sector. Our findings similarly indicate that the emotional impact of temperature varies across different groups. Specifically, we observed that women, unhealthy individuals, and rural residents are more susceptible to the influence of high temperatures on their mood.

Third, this study is closely connected with the impact of weather on cognitive performance. Previous studies have suggested that hostile weather conditions may reduce individuals’ cognitive performance (e.g.,^36,37^; a few exceptions include^38^ and^39^), and the effect may not be linear (e.g.,^40,41^). However, the question remains whether the impact of weather on cognitive performance is attributed solely to the strong association between mood and cognitive process (Dalgleish and Power^42^; for a recent review, see^43^). While some studies have supported this argument (e.g.,^44^), others have suggested that weather can simultaneously affect both mood and cognitive capacity, but independently^8,45^. Our analysis of the CHNS dataset does not yield evidence to substantiate the idea that temperature affects survey respondents’ cognitive capacity, despite the fact that their subjective evaluations are indeed influenced.

## Results

### The impact of temperature on self-reported income

We collect the individual-level income data from the China Health and Nutrition Survey (CHNS). The sampling frame is set up using a multistage, random cluster process to draw a sample of approximately 7200 households with more than 30,000 individuals in 15 provinces and municipal cities that vary substantially in geography, economic development, public resources, and health indicators. We match the CHNS data with the weather data from the Global Surface Summary of the Day (GSOD). The database encompasses a collection of over 800 weather stations operated by the China Meteorological Administration (CMA), among which 371 stations have weather records during the time window of the CHNS sample. Figure S1 illustrates the provinces participated in CHNS and the weather stations contained in GSOD. To isolate the weather effect on self-reporting activities from real income changes, we employ the disparity between the weather conditions on the interview date and the corresponding weekly averages as our weather metrics, which are the main predictors in our research. More details regarding the data and the econometric method are in Data and Methodology.

To examine the impact of weather conditions on self-reporting behavior, we first restrict our sample to individuals whose income information is entirely based on self-reporting. Table 1 column (1) shows that with a one-degree upward deviation from the weekly average temperature, a representative agent will underreport his or her income from the previous year by 81.09 RMB. To obtain a better understanding of the coefficient's magnitude, it is worth considering the temperature fluctuations within the specified time window and geographical location as quantified by the standard deviation of $\mathrm{\Delta temp}$, which amount to approximately 3.58 degrees Fahrenheit. Consequently, the benchmark outcome indicates that a respondent will report 581 RMB less on a hot day (e.g., one standard deviation above the mean) relative to a cold day (e.g., one standard deviation below the mean). The impact is statistically significant at the 5 percent level. The estimator is similar when using the weighted average weather record, as illustrated in column (2), or the subsample of counties with at least one weather station located within a 30-mile distance, as illustrated in column (3). To avoid overidentification, we also utilize the original weather metrics, namely daily average weather conditions, while excluding certain time-varying characteristics. The results, as shown in Table S2, are consistent in essence with those presented in Table 1.

**Table 1:** The impact of daily deviation from the weekly average temperature on self-reported annual income from the previous year.

	(1)	(2)	(3)
Outcome: self-reported net annual income from the previous year			
Weather station (WS) where the weather data is extracted from	Closest WS $\le$ 50 miles	Weighted weather data from WSs $\le$ 50 miles	Closest WS $\le$ 30 miles
$\mathrm{\Delta temp}$	− 81.09**	− 96.99***	− 101.8**
	*p* = 0.018	*p* = 0.009	*p* = 0.011
	(33.11)	(35.87)	(38.63)
Mean of outcome variable	9545.0	9494.2	9120.4
N	43,688	43,500	33,483

*Source* Panels of the China Health and Nutrition Survey (CHNS) from 1989 to 2015.

The sample is confined to individuals aged 16 and above whose income information are entirely self-reported. The key variable of interest is the daily temperature deviation of the survey date from the weekly average. The control variables include individual-level demographic and SES variables, the county-level indices, weather factors other than temperature, the weekly average temperature and other weather factors, individual fixed effects, and time fixed effects including year, month and weekday fixed effects. Standard errors are clustered at the county level. * significant at 10%. ** significant at 5%. *** significant at 1%.

### The non-linear influence of temperature

Numerous extant works propose the potential non-linear influence of temperature on individual behavior, as evidenced by studies such as^16,40^, and^41^, among others. In order to achieve a more comprehensive understanding of this relationship, we employ the panel data threshold regression model proposed by Hansen^46^ to investigate potential non-linearities. The estimations of cutoff points and their corresponding confidence intervals are enumerated in Panel A of Table S3. When applying a single threshold model, the temperature threshold is identified at 77 °F. In the case of adopting the double threshold model, the two threshold points align closely with that of the single threshold model. Alternatively, by considering the presence of an additional threshold, a value of 42.2 °F is derived. Regarding model selection, both the F-statistics shown in Panel B of Table S3 and the Hansen’s LR-statistics presented in Fig. S2 lend support to the adoption of the single threshold model.

We select the threshold of 77 °F to assess whether temperature variations might induce distinct impacts on self-reporting. The results are reported in Table 2 columns (1)–(3). We ascertain a modest yet statistically significant impact when the daily average temperature resides below 77 °F. Notably, the magnitude of impact experiences a marked escalation once temperatures surpass 77 °F. For conservativeness, we also explore the alternative cutoff of 42.2 °F and report the results in Table 2 columns (4)–(6). The results are largely the same, albeit with the distinction that the coefficient is positive but statistically insignificant for temperatures below 42.2 °F. To summarize, our results do not reveal a strictly U-shaped relationship, as evidenced in many previous studies. This divergence could stem from either the insignificant effect observed on comparatively colder days or the possibility that our regression outcomes predominantly capture the right side of the U-shaped curve. Nevertheless, our conclusions converge with prior research in the aspect that coefficients corresponding to relatively hot days exhibit markedly greater magnitudes in comparison to the average effects elucidated in Table 1.

**Table 2:** The non-linearity of the impact of temperature increase on self-reported annual income.

	(1)	(2)	(3)	(4)	(5)	(6)
Outcome: self-reported net annual income from the previous year						
Weather station (WS) where the weather data is extracted from	Closest WS $\le$ 50 miles	Weighted weather data from WSs $\le$ 50 miles	Closest WS $\le$ 30 miles	Closest WS $\le$ 50 miles	Weighted weather data from WSs $\le$ 50 miles	Closest WS $\le$ 30 miles
$\mathrm{\Delta temp}\times ({\text{Temperature}}<77.0\,\mathrm{^\circ{\rm F} })$	− 65.13*	− 79.34*	− 85.71**			
	*p* = 0.078	*p* = 0.051	*p* = 0.047			
	(36.25)	(39.64)	(41.94)			
$\mathrm{\Delta temp}\times ({\text{Temperature}}\ge 77.0\,\mathrm{^\circ{\rm F} })$	− 309.7**	− 349.5**	− 347.9**			
	*p* = 0.028	*p* = 0.012	*p* = 0.033			
	(137.4)	(135.0)	(158.5)			
$\mathrm{\Delta temp}\times ({\text{Temperature}}<42.2\,\mathrm{^\circ{\rm F} })$				73.57	68.78	59.02
				p = 0.266	p = 0.292	p = 0.341
				(65.45)	(64.64)	(61.36)
$\mathrm{\Delta temp}\times (42.2\mathrm{^\circ{\rm F} }\le {\text{Temperature}}<77.0\,\mathrm{^\circ{\rm F} })$				− 88.23*	− 103.7**	− 112.7**
				*p* = 0.053	*p* = 0.043	*p* = 0.038
				(44.56)	(50.03)	(52.82)
$\mathrm{\Delta temp}\times ({\text{Temperature}}\ge 77.0\,\mathrm{^\circ{\rm F} })$				− 307.4**	− 348.0**	− 346.6**
				*p* = 0.029	*p* = 0.013	*p* = 0.033
				(137.1)	(134.8)	(157.6)
Mean of outcome variable	9545.0	9494.2	9120.4	9545.0	9494.2	9120.4
N	43,688	43,500	33,483	43,688	43,500	33,483

*Source* Panels of the China Health and Nutrition Survey (CHNS) from 1989 to 2015.

The sample is confined to individuals aged 16 and above whose income information are entirely self-reported. The regime-dependent variable is the daily temperature deviation from the weekly average, and the threshold variable is the daily temperature. The control variables include individual-level demographic and SES variables, the county-level indices, weather factors other than temperature, the weekly average temperature and other weather factors, individual fixed effects, and time fixed effects. Standard errors are clustered at the county level. * significant at 10%. ** significant at 5%. *** significant at 1%.

### Placebo tests

As another attempt to rule out the possibility that the weather conditions captured on the survey date may reflect broader climate change patterns and thus impact real income, we conduct two placebo tests.

First, we estimate the impacts of the temperature one day before and one day after the interview date. Table S4 presents the results. Columns (1)–(3) show that the temperature one day before the interview has no impact on self-reported income. Columns (4)–(6) find the same results for the temperature one day after the interview. To eliminate the possibility that the insignificance is caused by the non-linearity proposed in Table 2, we also conduct the threshold regression model in columns (7) and (8), whose outcomes largely mirror those derived using the linear model. This is consistent with the conjecture that historical incomes reported by CHNS respondents only respond to the transient weather variations on the exact date of the interview.

In the second placebo test, we use imputed income as a placebo outcome. In the CHNS, imputation is performed when a respondent is determined to have income from a certain source, but the data of the exact income are incomplete.The resulting data are identified by a binary variable “Imputed”, which takes the value of 1 if the income data are partially/entirely derived from imputation instead of entirely based on the respondent’s self-reporting. Then, the CHNS will predict the annual income based on the average income from previous and subsequent waves and on community-level information. The imputed value is expected to be minimally impacted, if not entirely unaffected, by the weather on the survey date. This resilience arises from its reduced vulnerability to measurement errors associated with respondents' reporting activities, albeit potentially at the expense of a slightly elevated error level resulting from subsequent data cleaning procedures. We use this subsample of individuals with imputed income to fit Eq. (1) and replace the outcome with the imputed income. As illustrated in columns (1)–(3) of Table S5, the survey date temperature shows no statistically significant impact on the imputed income.

However, it should be acknowledged that because of the smaller sample size, controlling individual fixed effects is not feasible. To adopt a consistent specification, we exploit a larger sample including all individuals with either self-reported or imputed incomes. We differentiate the temperature effect on imputed and self-reported income using interactions. As can be observed in columns (4)–(6) of Table S5, the weather effect is statistically significant only if the income data are entirely reported by the respondents. We also conduct the threshold regression model in column (7), whose outcomes largely mirror those derived using the linear model.

Additionally, we investigate whether the absence of income information and the subsequent need for imputation are related to weather conditions on the survey date, to minimize the potential for selection contamination. The results are presented in Table S6 and demonstrate that there is no discernible relationship between the survey date temperature and the likelihood of income information imputation.

## Discussion

The classical survey response model^47^ breaks down response activity into four cognitive processes of understanding, retrieving, making judgments, and selecting a final response. While our research is not designed to explore the complete mechanisms of how weather affects the aforementioned processes due to data limitations, we aim to offer suggestive evidence regarding the impact of weather on respondents’ mood and cognitive capacity, two potential factors that could influence respondents’ cognitive performance.

We first examine the impact of weather on respondents’ mood, which we refer to as an affective state lasting longer than emotions but shorter than traits, typically enduring for hours or days^48^. Moods are commonly understood to alter information-processing priorities or bias cognitive processes, especially the memory retrieval process (e.g.,^11–13,49^; see^50^ for a review). Most weather parameters, including but not limited to wind power, sunlight, precipitation, air pressure, and photoperiod, are documented to have both unique and shared effects on individuals’ mood^9^. The mood-congruent effect hypothesis suggests that individuals tend to recall memories associated with similar emotional states. Thus, if higher temperature affects mood in a negative way (e.g.,^16,51,52^), a negatively affected mood may remind respondents of unpleasant experiences and make them pessimistic about their income.

Since the 2006 wave, the CHNS has introduced questions about respondents’ subjective judgments of life satisfaction (on a scale from 1 to 5). Respondents are further asked to give scale judgments regarding their recent moods with the following three statements: “I feel as energetic as before”, “I am as happy as before”, and “life is better than I thought”. Although the participation rate for the three subsequent questions is only 60% among those who report their life satisfaction, the evaluation of life satisfaction is mandatory. As proposed by Schwarz and Strack^53^, “respondents use their affective state at the time of judgment as a parsimonious indicator of their well-being in general.” Barrington-Leigh and Behzadnejad^7^ also argued that “the judgments of well-being measured by life satisfaction are partly informed by, or sensitive to, recent transitory factors such as mood.” This can also be demonstrated by Table S7, which clearly exhibits a significant correlation between life satisfaction and the responses to the three subsequent mood-related questions. Therefore, we choose life satisfaction as a proxy to examine whether temperature affects the respondents’ moods. Given the categorical nature of this variable, we fit the data with an ordered probit model and present the results in Panel B of Table 3.

**Table 3:** The heterogeneity of the temperature impact across individuals of different sex, BMI and residency area.

Panel A: The temperature impact on self-reported net annual income from the previous year							
	(1)	(2)	(3)	(4)	(5)	(6)	(7)
Sample	All	Female	Male	Abnormal BMI	Normal BMI	Rural	Urban
$\mathrm{\Delta temp}$	− 81.09**	− 104.5***	− 54.58	− 198.0**	− 11.57	− 67.09**	− 118.8
	*p* = 0.018	*p* = 0.010	*p* = 0.273	*p* = 0.035	*p* = 0.783	*p* = 0.037	*p* = 0.243
	(33.11)	(38.84)	(49.26)	(91.41)	(41.72)	(31.34)	(100.7)
Mean of outcome variable	9545.0	7805.9	11,116.1	12,192.1	8500.6	8142.0	13,486.1
N	43,688	20,735	22,953	12,361	31,327	32,218	11,470

Panel B: The temperature impact on life satisfaction							
	(8)	(9)	(10)	(11)	(12)	(13)	(14)
Sample	All	Female	Male	Abnormal BMI	Normal BMI	Rural	Urban
$\mathrm{\Delta temp}$	− 0.00700*	− 0.00989*	− 0.00520	− 0.0117**	− 0.00376	− 0.0112*	0.00000413
	*p* = 0.087	*p* = 0.085	*p* = 0.235	*p* = 0.044	*p* = 0.369	*p* = 0.055	*p* = 1.000
	(0.00409)	(0.00574)	(0.00438)	(0.00583)	(0.00419)	(0.00583)	(0.00874)
Mean of outcome variable	3.620	3.614	3.624	3.680	3.585	3.572	3.763
N	14,641	6551	8090	5366	9275	10,962	3679

Panel C: The temperature impact on questionnaire completeness							
	(15)	(16)	(17)	(18)	(19)	(20)	(21)
Sample	All	Female	Male	Abnormal BMI	Normal BMI	Rural	Urban
$\mathrm{\Delta temp}$	0.0000851	− 0.000224	0.000323	0.000330	− 0.0000318	0.000308	− 0.000315
	*p* = 0.897	*p* = 0.789	*p* = 0.611	*p* = 0.708	*p* = 0.966	*p* = 0.686	*p* = 0.723
	(0.000655)	(0.000830)	(0.000631)	(0.000879)	(0.000753)	(0.000756)	(0.000884)
Mean of outcome variable	21.97	21.97	21.97	21.97	21.97	21.97	21.97
N	51,063	24,283	26,780	14,481	36,582	37,989	13,074

*Source* Panels of the China Health and Nutrition Survey (CHNS) from 1989 to 2015.

The sample is confined to individuals aged 16 and above. The dependent variables in Panels A—C are the self-reported net annual income from the previous year, the life satisfaction, and the questionnaire completeness of the health and medical service survey, accordingly, in which life satisfaction data has been incorporated into the CHNS since the 2006 survey wave. The key variable of interest is the daily temperature deviation of the survey date from the weekly average. The weather data is extracted from the closest weather station within 50 miles from the county center. The “abnormal BMI” subsample contains individuals whose BMI $\ge 25$ or BMI $<18.5$, and the “normal BMI” subsample contains individuals with $18.5\le$ BMI $<25$. The control variables include individual-level demographic and SES variables, the county-level indices, weather factors other than temperature, the weekly average temperature and other weather factors, individual fixed effects, and time fixed effects. We adopt an OLS model in Panels A and C, and an ordered Probit model in Panel B. Standard errors are clustered at the county level. *significant at 10%. **significant at 5%. ***significant at 1%.

The findings are consistent with the traditional literature documenting that temperature pervasively affects individuals’ moods. Column (8) of Table 3 reveals that the daily variation in temperature has a significant negative impact on the life satisfaction evaluation.

As a sensitivity check, we exploit the perceived stress test provided only in the 2015 wave of CHNS. In Table S8, we show that higher temperatures result in increased reports of being upset, uncontrolled and overwhelmed among respondents, which is consistent with the observation in column (8) of Table 3. Furthermore, we address concerns regarding selection bias and present the corresponding results in Tables S9 and S10. Specifically, Table S9 reveals no significant relationship between the temperature on the survey date and the likelihood of reporting life satisfaction. Additionally, Table S10 suggests that narrowing down the sample to individuals who have ever engaged in the life satisfaction questionnaire will not alter the results in Table 1. This indicates that the difference in sample sizes between column (8) of Table 3 and Table 1 arises mainly from the absence of the life satisfaction questionnaire in the CHNS dataset before 2006, rather than disparities in the samples of surveyed individuals.

The existing body of literature provides rich implications for further investigating the potential heterogeneous impacts of high temperatures on mood. For instance, women and unhealthy individuals are pervasively reported to be more vulnerable to the effects of weather conditions (e.g.,^14–17^). Another example is that residents living in urban areas may have greater freedom to adjust to extreme temperatures, and surveys conducted in these areas are more likely to be conducted indoors, which eliminates potential weather effects (e.g.,^8,54^; an alternative explanation is the effect of memory anchoring, which may vary among individuals based on their unique experiences, e.g.,^55,56^).

Table 3 columns (9) and (10) show that women are more sensitive to weather conditions. Columns (11) and (12) show that the survey date temperature only has an effect on life satisfaction for the unhealthy subgroup, in which we adopt the body mass index (BMI) as a rule of thumb to categorize individuals as unhealthy or not. Finally, the survey date temperatures only have an effect on the life satisfaction of rural respondents, as presented in columns (13) and (14).

Panel A of Table 3 follows the separation of subgroups in Panel B while focusing on self-reported income as the dependent variable. The weather effects on self-reported income are only statistically significant for women, unhealthy people, and rural residents. The results perfectly match the heterogeneity observed in Panel B, providing supportive evidence that temperature causes systematic downward reporting of annual income from the previous year by affecting mood on the survey date.

In addition to the aforementioned heterogeneity analysis, we also examine the relationship between mood (proxied by life satisfaction) and self-reported income. Due to potential endogeneity issues in directly assessing their correlation, we adopt two alternative approaches. In Table S11, column (1), we employ the two-stage least squares (2SLS) approach by first estimating life satisfaction using temperature deviation, which ensures that this estimation reflects fluctuations solely attributable to mood changes triggered by temperature variations. Subsequently, we examine the correlation between the estimated life satisfaction and self-reported income, revealing a significant positive correlation. Our second test follows Baron and Kenny’s mediation analysis^57^. Specifically, we incorporate life satisfaction as an explanatory variable into the model in Table S10, revealing a significant positive coefficient. Notably, the significant correlation between weather conditions and self-reported income disappears, suggesting a considerable mediating effect of mood.

Before delving into cognitive capacity, it’s crucial to acknowledge that variations in interviewers’ characteristics might also contribute to respondents’ reporting biases. Regrettably, the CHNS doesn’t provide interviewer identifications, preventing us from incorporating interviewer fixed effects into our regressions. Nevertheless, we think this concern is largely assuaged by the fact that the matches of interviewers and interviewees are random. The perfect consistency of the impact on mood and on self-report bias across interviewees of different characteristics obviously does not depend on the traits of the assigned interviewers.

We subsequently investigate the influence of weather on respondents’ cognitive capacity, defined as the overall mental ability for performing cognitive tasks, which is typically assumed to be relatively autonomous from non-cognitive capacities such as affects or motivations^58^. Cognitive capacity sets limits on the extent to which individuals can engage in cognitive processes, thus determining their final cognitive performance. However, evidence regarding how temperature affects cognitive capacity is mixed in the literature (e.g.,^8,44,45^). If high temperatures indeed lower cognitive capacity, respondents may not accurately report their incomes.

Although the CHNS dataset lacks direct measures of respondents’ cognitive capacity, we use questionnaire completeness as a proxy. (Cognitive screen tests have been administered as part of the CHNS. However, these tests were only conducted in the surveys of 1997, 2000, 2004, and 2006, exclusively among individuals aged 55 years and above. This sample substantially diverges from our main study cohort.) Questionnaire completeness is believed to be determined by several interrelated dimensions, with primary factors including question features that affect respondent task complexity, and respondent characteristics including motivation, knowledge, and cognitive capacity (e.g.,^18,59–61^). Following Jeong et al.^18^, we construct the questionnaire completeness metric using a set of questions within the CHNS dataset characterized by specific criteria. These questions are mandatory for all participants across each survey iteration, maintain consistent content throughout the survey waves, and exclude queries related to medical insurance and expenditures to eliminate the possibility of respondents lacking relevant knowledge. Accordingly, we select two distinct sets of questions for calculating questionnaire completeness. One of these questionnaires, known as the healthcare and medical service survey, was introduced in the 1989 wave with a focus on gathering information about participants’ present health status, including experienced physical symptoms and recent medical services utilized. The second mandatory survey, introduced in the 1997 wave, focused on smoking, alcohol consumption, and physical activities, requiring participants to report their habits in these domains. We calculate questionnaire completeness by tallying the total number of answered questions in these two sets, excluding those marked as “unknown”. Such an approach can, to some extent, help isolate the impact of respondents’ cognitive capacity on questionnaire completeness. First, the consistent questionnaire content, along with year fixed effects controlled in our panel data analysis, ensures uniformity in question features across observations. Second, since these questions are mandatory, influence from respondent motivation is largely mitigated. Last, our focus solely on questions concerning respondents’ personal experiences and habits minimizes the impact of knowledge differences across respondents. Thus, we posit that the variation observed in this measure primarily stems from differences in respondents’ cognitive capacity.

Panel C of Table 3 shows the impact of the survey date temperatures on questionnaire completeness. We use the panels of the CHNS from 1989 to 2015. The dependent variable is the completeness of the healthcare and medical service survey. We also perform a replication exercise by limiting our sample to the CHNS waves from 1997 to 2015 and examining the completeness of the two aforementioned question sets. The findings, which closely mirror those in Panel C of Table 3, are presented in Table S13. Furthermore, we replicate the results in Panel C of Table 3 and Table S13 by adopting the threshold regression model to eliminate the possibility that the insignificance is caused by the non-linearity proposed in Table 2. The results are presented in Tables S14 and S15, whose outcomes largely mirror those derived using the linear model.

Table 3, column (15) shows that temperature has no effect on questionnaire completeness. Columns (16)–(21) show that neither of the subpopulations is responsive to the increase in temperature. These results presented in Panel C are consistent with our thought that if temperature lowers cognition capacity, we would expect a larger standard deviation of self-reported income but not a systematic downward bias.

To examine the validity of our constructed proxy, we replicate our questionnaire completeness measure in the China Family Panel Studies (CFPS) dataset, which includes four objective cognition tests of the respondent’s cognitive ability: the math test, the word test, the immediate word recall test and the delayed word recall test. The last two tests are taken from the Health and Retirement Study (HRS) conducted by the University of Michigan. Our completeness measure in the CFPS dataset comprises 11 mandatory questions that cover topics almost identical to those in the CHNS healthcare and medical service survey and the CHNS smoking, alcohol drinking, and physical activities survey, including the respondent’s current health condition, medical services received, smoking and drinking habits, and daily physical exercises. In Table S12, we show that questionnaire completeness in CFPS is significantly correlated with the respondent’s performance on immediate memory tests, math tests, word tests, and interviewer evaluations of the respondent’s intelligence.

Another caveat to consider is that our proxy should be viewed as related to, but not an exact measure of, the respondents' cognitive capacity. The results do not account for the potential influence of the interviewer’s characteristics, similar to the analysis of mood. Additionally, the measure does not completely eliminate respondent motivation, as they may intentionally choose not to answer questions to expedite the end of the survey, thus saving time, a phenomenon known as “satisficing”^59^. However, “satisficing” is generally believed to occur due to the excessive cognitive burden imposed by the survey, indicating that cognitive capacity still plays a role.

One final note is that our study remains silent regarding a potentially more fundamental mechanism, namely respondents’ health. It is known that respondents’ physical conditions can influence both mood and cognitive capacity. For instance, Pilgrim et al.^62^ suggests that blood pressure can affect mood, while fatigue is generally believed to affect cognitive capacity. Although the CHNS contains some health-related measures, such as those in Table S16 where we examined the impact of weather conditions on respondents’ blood pressure, our results, even if partially significant, lack robustness. The same phenomenon occurs in the examination of other health variables. One major reason for this is that health data in the CHNS may not always be collected on the same day as the questionnaire data, and the specific collection time is not disclosed. As a result, we are unable to accurately align health data with weather data from NOAA. Exploration of this mechanism awaits future research.

## Conclusions

This study investigates the influence of weather on reporting behavior. Our results demonstrate a statistically and economically significant reduction in reported annual income for the preceding year as the survey day's temperature rises, particularly on hotter days. Additionally, we employ heterogeneity analyses to delve into the pathways by which temperature impacts self-reporting behavior. We find that individuals more susceptible to mood changes induced by temperature are more inclined to underreport their income. This suggests that mood plays a pivotal role in how temperature affects reporting behavior, while no significant effects on cognitive capacity are detected in any subgroup. Our findings highlight the potential impact of temperature on survey data quality, underscoring the necessity for improved survey designs to mitigate weather-related biases. Moreover, further researches are needed to better understand the psychological and physiological mechanisms underpinning the influence of weather on income reporting.

## Data and methodology

### Data availability

Our study incorporates data from three publicly accessible datasets. For the original data, the individual-level data from CHNS can be accessed by the public after registering at www.cpc.unc.edu/projects/china, and the access to the community-level data from CHNS is granted upon approval at the same website. The GSOD data is freely available to the public and can be obtained at www.ncdc.noaa.gov. To access the individual-level data from CFPS, registration is required at www.isss.pku.edu.cn/cfps/. For those interested in requesting the matched data from this study, please contact Wang at xiangw@ruc.edu.cn.

### Sampling and variables of the China Health and Nutrition Survey (CHNS)

The research relies on data from two main sources. First, the individual-level self-reported and imputed income data and individual characteristics are from the China Health and Nutrition Survey (CHNS).CHNS is an ongoing international collaborative project between the Carolina Population Center at the University of North Carolina at Chapel Hill and the National Institute of Nutrition and Food Safety at the Chinese Center for Disease Control and Prevention. The sampling frame is set up using a multistage, random cluster process to draw a sample of approximately 7200 households with more than 30,000 individuals in 15 provinces and municipal cities that vary substantially in geography, economic development, public resources, and health indicators.

The provinces involved in this project include northeastern provinces such as Heilongjiang and Liaoning, middle areas such as Shandong and Henan, southern areas including Jiangsu, Hubei, Hunan and western areas including Guizhou and Guangxi.Shaanxi, Zhejiang and Yunnan were newly introduced in 2015 and thus are not included in the research. Since 1989, the project has conducted an unbalanced household panel survey, releasing data in ten publicly available waves: 1989, 1991, 1993, 1997, 2000, 2004, 2006, 2009, 2011, and 2015. The current survey took place over a seven-day period. The survey time is not fixed for each area. However, for the convenience of conducting interviews, the majority of surveys took place during the summer or when the weather is relatively mild. The respondents were interviewed with survey questionnaires and physical exams.

The CHNS aims to collect information on social and economic status (SES) and health and nutritional conditions. In the CHNS, individual net income is defined as the sum of seven potential sources of income: business, farming, fishing, gardening, livestock, nonretirement wages, and retirement income, with subsidies, gifts, rent, and in-kind payments excluded from the calculation. (According to the official documents of CHNS, “while household income includes the income from subsidies and other income, these cannot be allocated to individuals in the household and are not considered part of individual income.”) In cases where a respondent is determined to have income from a source based on filter questions or longitudinal files but the data are incomplete, an imputation is made using the individual’s reports from previous and subsequent waves or community-level information. Therefore, if a respondent reports having income in CHNS, the income information may be entirely based on the respondent’s self-reporting or partially/entirely derived from imputation. Our sample is composed of individuals aged 16 and above.

The CHNS has rich information that allows us to investigate the channels behind misreporting. In addition to regular questionnaires and physical exams that focus on objective biological and socioeconomic characteristics of the interviewees, the CHNS includes questions reflecting mood and mental health. Specifically, a problem set of psychological well-being was introduced into the CHNS 2006 and subsequent waves in which the respondents were asked to evaluate their life satisfaction and happiness. A stress test was introduced in 2015 in which the respondents describe their feelings and thoughts about their daily lives.

Panels A and B of Table S1 present the summary statistics of the CHNS individual-level demographics and SES and the self-rating categorical data about mood and happiness, respectively. At the county level, a list of urbanization indices is illustrated in Panel C of Table S1.

### Weather data from the Global Surface Summary of the Day (GSOD)

The weather data are extracted from the Global Surface Summary of the Day (GSOD), which is publicly available from the website of the National Oceanic and Atmospheric Administration (www.ncdc.noaa.gov). The weather data are collected from weather stations managed by the meteorological administrations of countries engaged in the Global Telecommunication System (GTS), and these data are subsequently distributed through the GTS network. For instance, the database encompasses a collection of over 800 weather stations operated by the China Meteorological Administration (CMA), among which 371 stations have weather records during the time window of the CHNS sample. Most CHNS communities are covered with a reasonable number of weather stations. For each weather station, the GSOD provides information on the location and daily weather records, including the daily average temperature, visibility, precipitation, and pressure. To match the CHNS data, we collected the weather conditions from January 1989 to December 2015. Figure S1 illustrates the provinces participated in CHNS and the weather stations contained in GSOD.

The CHNS administrative data of the community enables us to recover the name of each corresponding county. To match weather records with the locations involved, we adopt the criteria in^29^, that is, we merge the CHNS data with the weather records from the closest station based on the coordinates via the great circle navigation formula.

The average distance between the county centers and the corresponding weather station is approximately 21 miles, with approximately 93% of weather records collected within 50 miles. When the criterion is reduced to 30 miles, approximately 25% of the observations are dropped. We replicate the estimation using this smaller sample as a sensitivity check. Panel D of Table S1 describes the main variables collected from the GSOD data.

Given the abundant number of weather stations, Connolly^5^ uses the average from all weather stations within the boundary of the corresponding community. In the circumstance of no coverage in certain communities, Feddersen et al.^6^ use the weighted average of the closest three stations as a proxy for the weather conditions in the corresponding community. An alternative approach is to adopt the European Centre for Medium-Term Weather Forecasting (ECMWF), which uses an inverse distance weighting interpolation technique to generate a weather grid globally. Examples that employ this methodology include Heyes and Saberian^4^ and Schlenker and Roberts^63^. We adopt^29^ because of the spatial and temporal coverage of ground-based meteorological stations in China is limited. Additionally, the time span covered by our sample renders the employment of the ECMWF approach unfeasible. As an additional measure to assess the robustness of our analysis, we follow^6^ to construct weighted weather factors. In line with their approach, we gather weather data from all stations located within a 50-mile radius of each county in the CHNS dataset. Subsequently, we compute the weighted average of these weather records using the inverse of the distances as the respective weights.

### Cognition test score data from the China Family Panel Studies (CFPS)

In the discussion section, we draw upon cognitive test scores sourced from the China Family Panel Studies (CFPS). This comprehensive dataset, initiated in 2010 and subsequently conducted biennially, represents a nationally representative survey encompassing Chinese families and individuals. The CFPS is financially supported by Peking University and executed by the university’s Institute of Social Science Survey. To ensure an accurate representation of Chinese society, the survey employs a meticulously designed multistage probability proportional to size sampling approach, accompanied by implicit stratification.

Within the CFPS dataset, we find a wealth of cognitive assessment tools at our disposal. These encompass four objective cognition tests, namely the math test, word test, immediate word recall test, and delayed word recall test. Notably, the latter two assessments are adapted from the Health and Retirement Study (HRS) conducted by the University of Michigan. The math and word tests were administered during the 2010, 2014, and 2018 survey waves, while the memory tests were conducted during the 2012, 2016, and 2020 waves. Additionally, in each survey wave, CFPS interviewers provide subjective evaluations of respondents' intelligence. Panel E of Table S1 presents the summary statistics of the CFPS variables used in our research.

### Statistical method

In the benchmark analysis, we apply an individual fixed effects model, which is specified as follows:

1 $$Y_{ijt} = \beta^{W} W_{jt} + \beta^{X} X_{it} + \beta^{Z} Z_{jt} + \eta_{t} + u_{i} + \epsilon_{ijt}$$

The dependent variable ${Y}_{ijt}$ is the self-reported net income of individual *i* living in location *j* at survey date *t*. Weather conditions in county *j* at date *t* are denoted as ${W}_{jt}$, and they are the main variables of interest. We include time-varying individual control variables ${X}_{it}$, such as basic demographics and socioeconomic status (SES). The county-level time-variant control ${Z}_{jt}$ includes urbanization indices, such as population density, scores of social services, education, sanitation, and diversity. ${\eta }_{t}$ and ${u}_{i}$ are time and individual fixed effects, respectively.

In CHNS, the respondent is supposed to report his or her income from all sources in the year before the survey year, which implicitly indicates that the temperature on the survey dates should not affect the historical real incomes of the respondents. (For example, the wave in 1989 asked all respondents about their total income, if any, in 1988.) However, it is still possible that the weather conditions across years could be auto-correlated, at least to some extent. For instance, long-term climate conditions have a significant impact on agricultural productivity, human health and well-being, and energy production and consumption, all of which are important determinants of economic growth. As a result, the observed weather effect might simply represent the correlation between long-term climate conditions and the respondents’ real incomes. To better isolate the weather effect on self-reporting activities from real income changes, we employ the disparity between the weather conditions on the interview date and the corresponding weekly averages as our weather metrics. This approach isolates the influence of temporary weather fluctuations, akin to a quasi-natural experiment^7^. We further control the month fixed effects, the weekday fixed effects and the weekly average weather conditions to eliminate possible seasonal variations and intraweek fluctuations. In addition, considering that unobservable factors might be correlated in a broader area than the community, we cluster the error term at the county level in all regressions. For the robustness check on non-linearity, we incorporate Hansen’s threshold regression^46^ into our benchmark model, and incorporate the methodology outlined in^64^ for model estimation.

### Supplementary Information

Supplementary Information.
