# Supplementary material for: The temperature effect on perceived income
Source: Sci Rep. 2024 Mar 14;14:6169. doi: 10.1038/s41598-024-56852-2 (PMC11344060; doi:10.1038/s41598-024-56852-2)
Supplement: Supplementary file 1 — Supplementary Information. [file 41598_2024_56852_MOESM1_ESM.docx]

**SI Appendix Supporting Figures and Tables for**

**The Temperature Effect on Perceived Income**

**Manuscript ID: 018307fe-e66e-43df-a5d8-3d1ee6deb574**

Ang Sun^a^, Wang Xiang^b^, Xu Jiang^c^

^a^School of Finance, Renmin University of China, [ang.sun@gmail.com](mailto:ang.sun@gmail.com)

^b^Corresponding author, School of Finance, Renmin University of China, [xiangw@ruc.edu.cn](mailto:xiangw@ruc.edu.cn)

^c^School of Finance, Renmin University of China, [xu.jiang@ruc.edu.cn](mailto:xu.jiang@ruc.edu.cn)

This file includes:

SI Appendix Supporting Figures and Tables

Figures S1 to S2

Tables S1 to S16

*Fig. S1. Provinces Participated in CHNS and Weather Stations in GSOD*


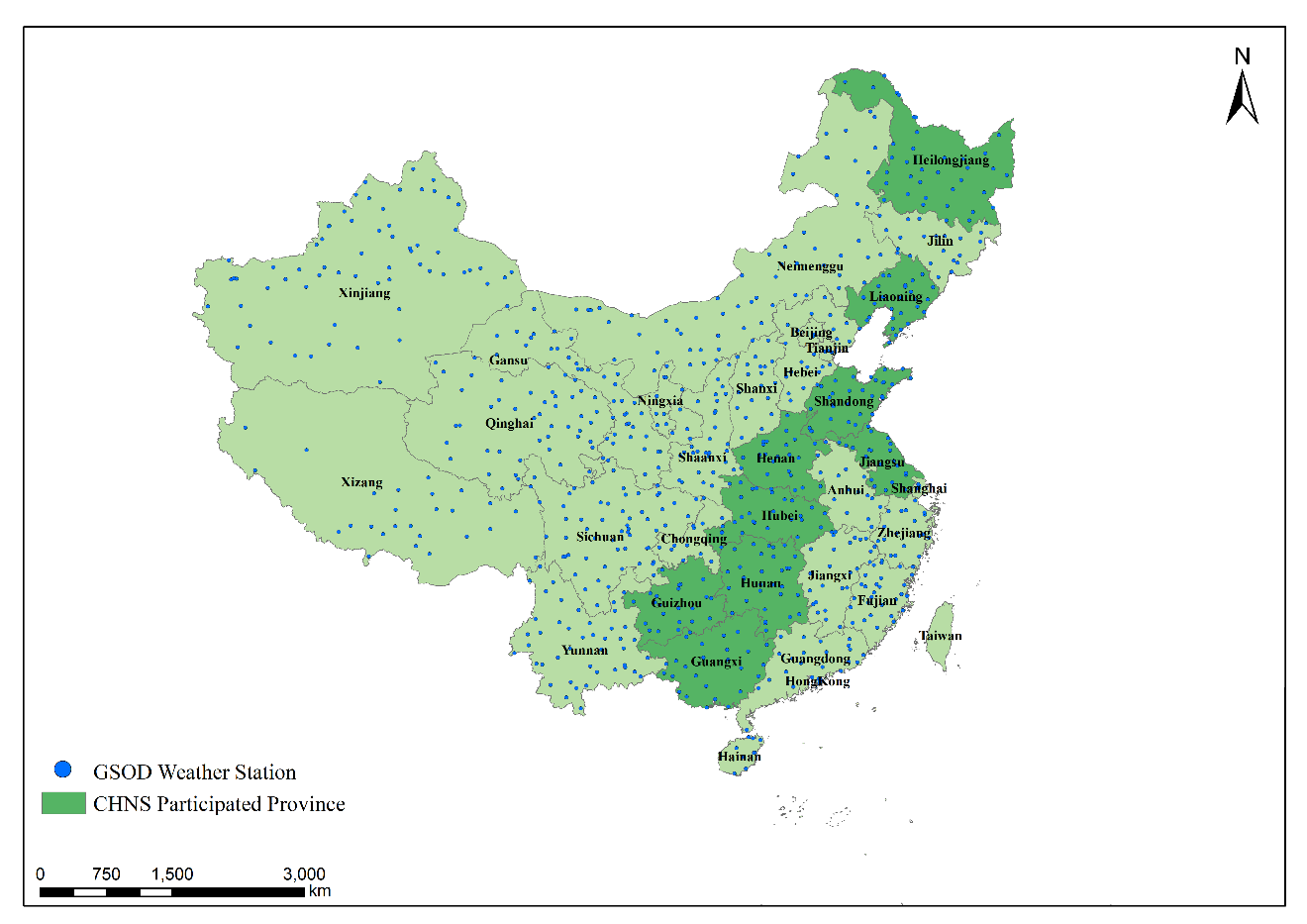


SOURCE - [**www.cpc.unc.edu/projects/china**](http://www.cpc.unc.edu/projects/china) and [**www.ncdc.noaa.gov**](http://www.ncdc.noaa.gov)

NOTE - Among these provinces, Shaanxi, Zhejiang and Yunnan were newly introduced in CHNS in 2015 and thus are not included in the research.

*Fig. S2. Hansen’s LR Statistics for the Threshold Regression*


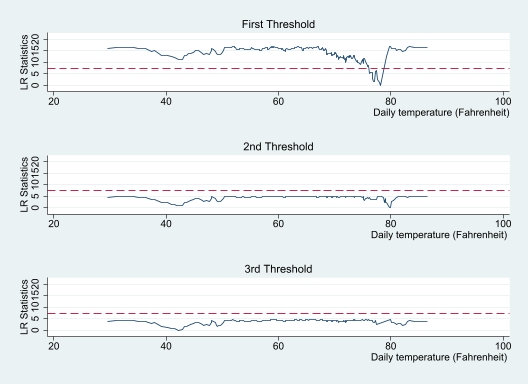


SOURCE - Panels of the China Health and Nutrition Survey (CHNS) from 1989 to 2015.

NOTE - The sample is confined to individuals aged 16 and above whose income information are entirely self-reported. The regime-dependent variable is the daily temperature deviation from the weekly average, and the threshold variable is the daily temperature. The control variables include individual-level demographic and SES variables, the county-level indices, weather factors other than temperature, the weekly average temperature and other weather factors, individual fixed effects, and time fixed effects.

*Table S1. Summary Statistics of the CHNS, the CFPS and the GSOD*

|  | Obs. | Mean | Std. Dev. | Min | Max |
| --- | --- | --- | --- | --- | --- |
| Panel A: CHNS Individual Demographic and SES Variables | | | | | |
| Individual total net income | 47054 | 9492.90 | 23550.69 | -312964 | 1142168.5 |
| - Entirely self-reported | 43896 | 9646.18 | 24115.51 | -312964 | 1142168.5 |
| - Partially/entirely imputed | 3158 | 7362.31 | 13251.68 | -103390 | 308004 |
| Married | 47054 | 0.8441 | 0.3627 | 0 | 1 |
| Education years | 47054 | 7.3714 | 4.1393 | 0 | 18 |
| Employed | 47054 | 0.9625 | 0.1899 | 0 | 1 |
| Is household head | 47054 | 0.4126 | 0.4923 | 0 | 1 |
| Is official cadre | 47054 | 0.0219 | 0.1463 | 0 | 1 |
| Is Five-Guaranteed family | 47054 | 0.0001 | 0.0103 | 0 | 1 |
| Male | 47054 | 0.5224 | 0.4995 | 0 | 1 |
| Age | 47054 | 42.3164 | 13.09 | 16 | 93 |
| Current health (1 if sick) | 47054 | 0.0940 | 0.2918 | 0 | 1 |
| Height (cm) | 47054 | 161.14 | 8.4610 | 48 | 192 |
| Weight (kg) | 47054 | 58.95 | 10.92 | 30 | 160 |
| Panel B: CHNS Mood Related Variables (5 represents highest level) | | | | | |
| Life satisfaction | 14866 | 3.6207 | 0.8218 | 1 | 5 |
| Happiness | 7915 | 3.1108 | 0.8695 | 1 | 5 |
| Level of energy | 7932 | 3.1088 | 0.8971 | 1 | 5 |
| Positivity | 7872 | 3.2918 | 0.8443 | 1 | 5 |
| Upset | 3219 | 2.5381 | 0.7537 | 1 | 5 |
| Uncontrolled | 3207 | 2.4521 | 0.7760 | 1 | 5 |
| Nervous and stressed | 3219 | 2.5971 | 0.7733 | 1 | 5 |
| Resilience | 3166 | 3.2189 | 0.9588 | 1 | 5 |
| Adjustment | 3083 | 3.1304 | 1.0080 | 1 | 5 |
| Confident | 3172 | 3.6680 | 0.9110 | 1 | 5 |
| Things were going your way | 3133 | 3.4392 | 0.9019 | 1 | 5 |
| Burdened | 3151 | 2.7331 | 0.8817 | 1 | 5 |
| Able to control irritations | 3171 | 3.4219 | 0.9364 | 1 | 5 |
| Capable | 3114 | 3.4891 | 0.9725 | 1 | 5 |
| Anger | 3211 | 2.5783 | 0.7474 | 1 | 5 |
| Preoccupation | 3133 | 2.7807 | 0.8765 | 1 | 5 |
| Scheduled | 3138 | 3.4101 | 0.9804 | 1 | 5 |
| Overwhelmed | 3137 | 2.5461 | 0.8139 | 1 | 5 |
| Panel C: CHNS County Level Indices | | | | | |
| Social services score | 47046 | 1.7098 | 2.5205 | 0 | 10 |
| Population density score | 47046 | 5.7713 | 1.3597 | 0 | 10 |
| Education score | 47046 | 3.0912 | 1.4671 | 0.48 | 8.80 |
| Sanitation score | 47046 | 5.7210 | 3.1341 | 0 | 10 |
| Urbanization index | 47046 | 55.84 | 19.68 | 16.75 | 106.50 |
| Diversity score | 47046 | 4.5313 | 1.2524 | 1.50 | 9.50 |
| Quality of health score | 47046 | 5.4590 | 2.3112 | 0 | 10 |
| Panel D: GSOD Weather Variables | | | | | |
| Temperature (Fahrenheit) | 47054 | 63.16 | 12.04 | -12.50 | 90.10 |
| Visibility (Miles) | 47054 | 8.2167 | 3.6455 | 0 | 18.60 |
| Sea level pressure (Mb) | 47054 | 48.00 | 66.63 | 29.37 | 295.30 |
| Precipitation (Inches) | 47053 | 0.0990 | 0.2958 | 0 | 4.02 |
| Station pressure (Mb) | 47054 | 21.93 | 12.29 | 0 | 29.53 |
| Panel E: CFPS Variables | | | | | |
| Instant memory test score | 60222 | 4.6690 | 1.9490 | 0 | 10 |
| Delayed memory test score | 59133 | 3.6660 | 2.2280 | 0 | 10 |
| Math test score | 98255 | 8.5640 | 7.8140 | -8 | 24 |
| Word test score | 98255 | 15.78 | 12.40 | -8 | 34 |
| Respondent’s intelligence | 190747 | 5.226 | 1.344 | 1 | 7 |
| Age | 191165 | 44.84 | 17.30 | 9 | 110 |
| Married | 186604 | 0.7850 | 0.4110 | 0 | 1 |
| Employed | 177147 | 0.6700 | 0.4700 | 0 | 1 |
| Education year | 183936 | 7.5040 | 4.8110 | 0 | 24 |

SOURCE - Panels of the China Health and Nutrition Survey (CHNS) from 1989 to 2015, and the corresponding weather data from the Global Summary of the Day (GSOD). Panels of the China Family Panel Studies (CFPS) from 2010 to 2020.

Note - The CHNS sample is confined to individuals aged 16 and above who are matched with the GSOD sample.

*Table S2. Robustness Check: The Impact of Daily Temperature on Self-reported Annual Income from the Previous Year*

|  | (1) | (2) | (3) | (4) | (5) |
| --- | --- | --- | --- | --- | --- |
| Outcome: Self-reported net annual income from the previous year | | | | | |
| Weather station (WS) where the  weather data is extracted from | Closest WS  $\leq$50 miles | Closest WS  $\leq$50 miles | Closest WS  $\leq$50 miles | Weighted weather  data from WSs  $\leq$50 miles | Closest WS  $\leq$30 miles |
| Temperature | -29.95** | -31.21** | -33.37** | -41.25*** | -26.28* |
|  | p=0.033 | p=0.029 | p=0.023 | p=0.006 | p=0.092 |
|  | (13.66) | (13.91) | (14.26) | (14.50) | (15.28) |
| County-level indices | No | Yes | Yes | Yes | Yes |
| Other weather factors | No | No | Yes | Yes | Yes |
| Mean of outcome variable | 9646.2 | 9641.3 | 9640.5 | 9631.3 | 9184.3 |
| N | 43896 | 43888 | 43887 | 43849 | 33534 |

SOURCE - Panels of the China Health and Nutrition Survey (CHNS) from 1989 to 2015.

NOTE - The sample is confined to individuals aged 16 and above whose income information are entirely self-reported. The key variable of interest is the temperature on the survey day. The control variables include individual fixed effects, year fixed effects, and the individual-level demographic and SES variables. We further control the county-level indices in columns (2) - (5), and weather factors other than temperature in columns (3) - (5). Standard errors are clustered at the county level. * significant at 10%. ** significant at 5%. *** significant at 1%.

*Table S3. The Threshold Regression of the Impact of Temperature Increase on Self-reported Annual Income*

| Panel A: Threshold estimator and 95% confidence interval | | | | | |
| --- | --- | --- | --- | --- | --- |
| Model |  | Threshold | Lower | Upper |  |
| Single threshold model - 1st threshold |  | 77.0 | 73.5 | 77.1 |  |
| Double threshold model - 1st threshold |  | 78.1 | 77.0 | 79.3 |  |
| Double threshold model - 2nd threshold |  | 79.9 | 79.7 | 80.2 |  |
| Multiple threshold model - 3rd threshold |  | 42.2 | 41.9 | 42.9 |  |
| Panel B: Threshold-effect test result | | | | | |
| Model | F-statistics | P-value | 10%  Critical Value | 5%  Critical Value | 1%  Critical Value |
| Single threshold model | 6.3682 | 0.086 | 6.1069 | 7.3422 | 10.6471 |
| Double threshold model | 3.6918 | 0.406 | 6.7633 | 8.1888 | 16.9565 |
| Multiple threshold model | 2.5258 | 0.814 | 10.4007 | 13.7808 | 20.9847 |

SOURCE - Panels of the China Health and Nutrition Survey (CHNS) from 1989 to 2015.

NOTE - The sample is confined to individuals aged 16 and above whose income information are entirely self-reported. We employ the algorithm in [64] to estimate the panel data threshold regression model proposed in Hansen ([46]) with 500 bootstraps. The regime-dependent variable is the daily temperature deviation from the weekly average, and the threshold variable is the daily temperature. The control variables include individual-level demographic and SES variables, the county-level indices, weather factors other than temperature, the weekly average temperature and other weather factors, individual fixed effects, and time fixed effects.

*Table S4. Placebo Test: The Impact of Daily Deviation of One Day Before and After the Survey Date from the Weekly Average Temperature*

|  | (1) | (2) | (3) | (4) | (5) | (6) | (7) | (8) |
| --- | --- | --- | --- | --- | --- | --- | --- | --- |
| Outcome: Self-reported net annual income from the previous year | | | | | | | | |
| Weather station (WS) where the  weather data is extracted from | Closest WS  $\leq$50 miles | Weighted weather  data from WSs  $\leq$50 miles | Closest WS  $\leq$30 miles | Closest WS  $\leq$50 miles | Weighted weather  data from WSs  $\leq$50 miles | Closest WS  $\leq$30 miles | Closest WS  $\leq$50 miles | Closest WS  $\leq$50 miles |
| $\Delta\mathrm{temp}_{t+1}$ | -40.51 | -33.98 | -66.01 |  |  |  |  |  |
|  | p=0.351 | p=0.493 | p=0.128 |  |  |  |  |  |
|  | (43.04) | (49.25) | (42.53) |  |  |  |  |  |
| $\Delta\mathrm{temp}_{t-1}$ |  |  |  | -35.78 | -47.96 | -19.39 |  |  |
|  |  |  |  | p=0.356 | p=0.252 | p=0.664 |  |  |
|  |  |  |  | (38.41) | (41.43) | (44.39) |  |  |
| $\Delta\mathrm{temp}_{t+1}\times(Temperature<77.0℉)$ |  |  |  |  |  |  | -42.30 |  |
|  |  |  |  |  |  |  | p=0.355 |  |
|  |  |  |  |  |  |  | (45.30) |  |
| $\Delta\mathrm{temp}_{t+1}\times(Temperature\geq77.0℉)$ |  |  |  |  |  |  | -13.49 |  |
|  |  |  |  |  |  |  | p=0.901 |  |
|  |  |  |  |  |  |  | (108.4) |  |
| $\Delta\mathrm{temp}_{t-1}\times(Temperature<77.0℉)$ |  |  |  |  |  |  |  | -44.31 |
|  |  |  |  |  |  |  |  | p=0.261 |
|  |  |  |  |  |  |  |  | (38.99) |
| $\Delta\mathrm{temp}_{t+1}\times(Temperature\geq77.0℉)$ |  |  |  |  |  |  |  | 133.4 |
|  |  |  |  |  |  |  |  | p=0.373 |
|  |  |  |  |  |  |  |  | (148.6) |
| Mean of outcome variable | 9545.0 | 9494.2 | 9120.4 | 9545.0 | 9494.2 | 9120.4 | 9545.0 | 9545.0 |
| N | 43688 | 43500 | 33483 | 43688 | 43500 | 33483 | 43688 | 43688 |

SOURCE - Panels of the China Health and Nutrition Survey (CHNS) from 1989 to 2015.

NOTE - The sample is confined to individuals aged 16 and above whose income information are entirely self-reported. The key variables of interest are the daily temperature deviation of one day before and after the survey date from the weekly average. The control variables include individual-level demographic and SES variables, the county-level indices, the weekly average temperature and other weather factors, individual fixed effects, and time fixed effects. Standard errors are clustered at the county level. For brevity, we solely present outcomes wherein meteorological data is sourced from the nearest station within a 50-mile radius in columns (7) and (8). The findings obtained using weighted weather data are largely the same. * significant at 10%. ** significant at 5%. *** significant at 1%.

*Table S5. Placebo test: The Impact of Daily Deviation from the Weekly Average Temperature on Imputed Annual Income from the Previous Year*

|  | (1) | (2) | (3) | (4) | (5) | (6) | (7) |
| --- | --- | --- | --- | --- | --- | --- | --- |
| Outcome: Net annual income from the previous year | | | | | | | |
| Sample is confined to | Imputed | Imputed | Imputed | All | All | All | All |
| Weather station (WS) where the  weather data is extracted from | Closest WS  $\leq$50 miles | Weighted weather  data from WSs  $\leq$50 miles | Closest WS  $\leq$30 miles | Closest WS  $\leq$50 miles | Weighted weather  data from WSs  $\leq$50 miles | Closest WS  $\leq$30 miles | Closest WS  $\leq$50 miles |
| $\Delta temp$ | -29.29 | -43.48 | -30.96 |  |  |  |  |
|  | p=0.558 | p=0.357 | p=0.548 |  |  |  |  |
|  | (49.70) | (46.81) | (51.17) |  |  |  |  |
| $\Delta\mathrm{temp}^{\mathrm{sr}}$ |  |  |  | -81.36** | -94.78*** | -103.8*** | -81.36** |
|  |  |  |  | p=0.016 | p=0.009 | p=0.009 | p=0.016 |
|  |  |  |  | (32.65) | (35.07) | (38.28) | (32.62) |
| $\Delta temp^{\mathrm{nsr}}$ |  |  |  | -58.74 | -80.19 | -61.61 |  |
|  |  |  |  | p=0.407 | p=0.272 | p=0.405 |  |
|  |  |  |  | (70.20) | (72.29) | (73.23) |  |
| $\Delta temp^{\mathrm{nsr}}\times(Temperature<77.0℉)$ |  |  |  |  |  |  | -58.70 |
|  |  |  |  |  |  |  | p=0.396 |
|  |  |  |  |  |  |  | (68.53) |
| $\Delta temp^{\mathrm{nsr}}\times(Temperature\geq77.0℉)$ |  |  |  |  |  |  | -59.35 |
|  |  |  |  |  |  |  | p=0.804 |
|  |  |  |  |  |  |  | (238.5) |
| Individual FE | No | No | No | Yes | Yes | Yes | Yes |
| Mean of outcome variable | 7327.5 | 7317.8 | 7312.2 | 9396.0 | 9347.5 | 8988.7 | 9396.0 |
| N | 3148 | 3145 | 2631 | 46836 | 46645 | 36114 | 46836 |

SOURCE - Panels of the China Health and Nutrition Survey (CHNS) from 1989 to 2015.

NOTE - The sample is confined to individuals aged 16 and above. In columns (1) - (3), the sample is further confined to individuals whose income information are partially/entirely imputed. The key variables of interest are the daily temperature deviation from the weekly average (columns (1) - (3)), the interactions $\Delta\mathrm{temp}^{\mathrm{sr}}=\Delta temp\times(Imputed=1)$ and $\Delta\mathrm{temp}^{\mathrm{nsr}}=\Delta temp\times(Imputed=0)$ where $Imputed$ is the dummy indicator of whether the net income information are partially/entirely imputed (columns (4) - (7)). The control variables include individual-level demographic and SES variables, the county-level indices, weather factors other than temperature, the weekly average temperature and other weather factors, and time fixed effects. Standard errors are clustered at the county level. For brevity, we solely present outcomes wherein meteorological data is sourced from the nearest station within a 50-mile radius in column (7). The findings obtained using weighted weather data are largely the same. * significant at 10%. ** significant at 5%. *** significant at 1%.

*Table S6. Robustness Check: The Impact of Temperature Deviation from the Weekly Average on the Incidence of Imputed Income*

|  | (1) | (2) | (3) |
| --- | --- | --- | --- |
| Outcome: Indicator of whether the income information is imputed | | | |
| Model | OLS | Probit | Logit |
| $\Delta temp$ | -0.000553 | -0.000358 | 0.000973 |
|  | p=0.564 | p=0.963 | p=0.954 |
|  | (0.000953) | (0.00773) | (0.0169) |
| Individual FE | Yes | No | No |
| Mean of outcome variable | 0.0672 | 0.0672 | 0.0672 |
| N | 46836 | 46789 | 46789 |

SOURCE - Panels of the China Health and Nutrition Survey (CHNS) from 1989 to 2015.

NOTE - The sample is confined to individuals aged 16 and above. The key variable of interest is the daily temperature deviation of the survey date from the weekly average. The weather data is extracted from the closest weather station within 50 miles from the county center. The control variables include individual-level demographic and SES variables, the county-level indices, weather factors other than temperature, the weekly average temperature and other weather factors, individual fixed effects, and time fixed effects. Standard errors are clustered at the county level. * significant at 10%. ** significant at 5%. *** significant at 1%.

*Table S7. The Association between Self-reported Life Satisfaction and Other Measures of Mood*

|  | (1) | (2) | (3) |
| --- | --- | --- | --- |
| Outcome: | Happiness | Level of energy | Positivity |
| Life satisfaction | 0.289*** | 0.193*** | 0.341*** |
|  | p<0.001 | p<0.001 | p<0.001 |
|  | (0.0364) | (0.0224) | (0.0254) |
| Mean of outcome variable | 3.110 | 3.108 | 3.291 |
| N | 7874 | 7891 | 7829 |

SOURCE - Panels of the China Health and Nutrition Survey (CHNS) from 2006 to 2015.

NOTE - The sample is confined to individuals aged 16 and above who are selected to answer questions regarding their psychological wellbeing. The key variable of interest is the life satisfaction. The control variables include individual-level demographic and SES variables, the county-level indices, and time fixed effects. We adopt an ordered Probit model and calculate standard errors clustered by county. * significant at 10%. ** significant at 5%. *** significant at 1%.

*Table S8. The Impact of Temperature Deviation from the Weekly Average on the Perceived Stress*

|  | (1) | (2) | (3) | (4) | (5) |
| --- | --- | --- | --- | --- | --- |
| Outcome: | Upset | Uncontrolled | Nervous | Resilience | Adjustment |
| $\Delta temp$ | 0.0173* | 0.0150* | 0.0117 | 0.00500 | 0.00696 |
|  | p=0.055 | p=0.073 | p=0.274 | p=0.569 | p=0.446 |
|  | (0.00904) | (0.00840) | (0.0107) | (0.00877) | (0.00913) |
| Mean of outcome | 2.641 | 2.580 | 2.699 | 3.404 | 3.468 |
| N | 3271 | 3271 | 3271 | 3271 | 3271 |
|  | (6) | (7) | (8) | (9) | (10) |
|  | Confident | Success | Burdened | Irritation-control | Capable |
| $\Delta temp$ | -0.00225 | 0.00148 | 0.0204** | 0.00959 | 0.00981 |
|  | p=0.826 | p=0.876 | p=0.025 | p=0.296 | p=0.330 |
|  | (0.0103) | (0.00952) | (0.00912) | (0.00917) | (0.0101) |
| Mean of outcome | 3.829 | 3.674 | 2.963 | 3.592 | 3.754 |
| N | 3271 | 3271 | 3271 | 3271 | 3271 |
|  | (11) | (12) | (13) | (14) |  |
|  | Anger | Preoccupation | Scheduled | Overwhelmed |  |
| $\Delta temp$ | 0.00331 | 0.00813 | -0.00894 | 0.0288*** |  |
|  | p=0.762 | p=0.485 | p=0.346 | p=0.008 |  |
|  | (0.0110) | (0.0116) | (0.00948) | (0.0108) |  |
| Mean of outcome | 2.696 | 3.043 | 3.637 | 2.810 |  |
| N | 3271 | 3271 | 3271 | 3271 |  |

SOURCE - The perceived stress test from 2015 China Health and Nutrition Survey (CHNS).

NOTE - The sample is confined to individuals aged 16 and above. The key variable of interest is the daily temperature deviation of the survey date from the weekly average. The weather data is extracted from the closest weather station within 50 miles from the county center. The control variables include individual-level demographic and SES variables, the county-level indices, weather factors other than temperature, the weekly average temperature and other weather factors, and time fixed effects including month and weekday fixed effects. We adopt an ordered Probit model and calculate standard errors clustered by county using the Wild Bootstrap method. * significant at 10%. ** significant at 5%. *** significant at 1%.

*Table S9. Robustness Check: The Impact of Temperature Deviation from the Weekly Average on the Incidence of Life Satisfaction Missing Values*

|  | (1) | (2) | (3) |
| --- | --- | --- | --- |
| Outcome: Indicator of whether life satisfaction is not missing | | | |
| Model | OLS | Probit | Logit |
| $\Delta temp$ | -0.000472 | 0.00520 | -0.000401 |
|  | p=0.428 | p=0.655 | p=0.981 |
|  | (0.00134) | (0.0116) | (0.0171) |
| Individual FE | Yes | No | No |
| Mean of outcome variable | 0.774 | 0.774 | 0.774 |
| N | 18906 | 18906 | 18906 |

SOURCE - Panels of the China Health and Nutrition Survey (CHNS) from 2006 to 2015.

NOTE - The sample is confined to individuals aged 16 and above. The key variable of interest is the daily temperature deviation of the survey date from the weekly average. The weather data is extracted from the closest weather station within 50 miles from the county center. The control variables include individual-level demographic and SES variables, the county-level indices, weather factors other than temperature, the weekly average temperature and other weather factors, and time fixed effects. Standard errors are clustered at the county level using the Wild Bootstrap method. * significant at 10%. ** significant at 5%. *** significant at 1%.

*Table S10. Robustness Check: The Impact of Daily Deviation from the Weekly Average Temperature on Self-reported Annual Income from the Previous Year, Contingent upon Respondents Who Have Engaged in the Life Satisfaction Survey*

|  | (1) | (2) | (3) |
| --- | --- | --- | --- |
| Outcome: Self-reported net annual income from the previous year | | | |
| Weather station (WS) where the weather data is extracted from | Closest WS  $\leq$50 miles | Weighted weather  data from WSs  $\leq$50 miles | Closest WS  $\leq$30 miles |
| $\Delta temp$ | -85.53** | -103.8*** | -102.7** |
|  | p=0.014 | p=0.007 | p=0.014 |
|  | (33.60) | (37.26) | (40.31) |
| Mean of outcome variable | 8391.3 | 8338.4 | 7907.2 |
| N | 41142 | 40967 | 31441 |

SOURCE - Panels of the China Health and Nutrition Survey (CHNS) from 1989 to 2015.

NOTE - The sample is confined to individuals aged 16 and above who have ever engaged in the life satisfaction survey since 2006, and whose income information are entirely self-reported. The key variable of interest is the daily temperature deviation of the survey date from the weekly average. The control variables include individual-level demographic and SES variables, the county-level indices, weather factors other than temperature, the weekly average temperature and other weather factors, individual fixed effects, and time fixed effects including year, month and weekday fixed effects. Standard errors are clustered at the county level. * significant at 10%. ** significant at 5%. *** significant at 1%.

*Table S11. Mediation Analysis: The Impact of Daily Deviation from the Weekly Average Temperature on Self-reported Annual Income from the Previous Year, with Life Satisfaction as the Mediating Variable*

|  | (1) | (2) |
| --- | --- | --- |
| Outcome: Self-reported net annual income from the previous year | | |
| Estimated life satisfaction | 152944.2** |  |
|  | p=0.026 |  |
|  | (66877.5) |  |
| $\Delta temp$ |  | 102.8 |
|  |  | p=0.495 |
|  |  | (149.5) |
| Life satisfaction |  | 1226.5* |
|  |  | p=0.083 |
|  |  | (694.3) |
| Mean of outcome variable | 8391.3 | 20703.9 |
| N | 41142 | 12670 |

SOURCE - Panels of the China Health and Nutrition Survey (CHNS) from 1989 to 2015.

NOTE - The sample is confined to individuals aged 16 and above who have ever engaged in the life satisfaction survey since 2006, and whose income information are entirely self-reported. The weather data is extracted from the closest weather station within 50 miles from the county center. The key variable of interest is the daily temperature deviation of the survey date from the weekly average, and the life satisfaction. The control variables include individual-level demographic and SES variables, the county-level indices, weather factors other than temperature, the weekly average temperature and other weather factors, individual fixed effects, and time fixed effects including year, month and weekday fixed effects. Standard errors are clustered at the county level. * significant at 10%. ** significant at 5%. *** significant at 1%.

*Table S12. Robustness Check: The Association between Questionnaire Completeness and Respondents Cognition (CFPS)*

|  | (1) | (2) | (3) | (4) | (5) |
| --- | --- | --- | --- | --- | --- |
| Outcome: | IWR score | DWR score | Math test | Word test | Intelligence |
| Completeness | 0.363*** | 0.196 | 0.978*** | 1.441*** | 0.0741** |
|  | p=0.001 | p=0.183 | p<0.001 | p<0.001 | p=0.038 |
|  | (0.109) | (0.147) | (0.0688) | (0.112) | (0.0357) |
| Mean of outcome variable | 4.669 | 3.666 | 8.564 | 15.78 | 5.226 |
| N | 54248 | 53218 | 89396 | 89396 | 169305 |

SOURCE - Panels of the China Family Panel Studies (CFPS) from 2010 to 2020.

NOTE - The dependent variables are the score of the immediate word recall test, the score of the delayed word recall test, the math test score, the word test score, and the interviewer’s subjective judgement of the respondent’s intelligence, respectively. The key variable of interest is the questionnaire completeness regarding the healthcare and diet problems in CFPS. The control variables include the respondent’s age, employment status, education year, marriage status, individual fixed effects, and year and month fixed effects. Standard errors are clustered at the county level. * significant at 10%. ** significant at 5%. *** significant at 1%.

*Table S13. Robustness Check: The Impact of Daily Deviation from the Weekly Average Temperature on Cognition Proxied by Questionnaire Completeness, including Questions about Diet and Physical Activities*

|  | (1) | (2) | (3) | (4) | (5) | (6) | (7) |
| --- | --- | --- | --- | --- | --- | --- | --- |
| Outcome: Questionnaire completeness of the healthcare and medical service survey and the smoking, alcohol drinking, and physical activities survey | | | | | | | |
| Sample | All | Female | Male | Abnormal BMI | Normal BMI | Rural | Urban |
| $\Delta temp$ | -0.0000174 | -0.000754 | 0.000600 | 0.00228 | -0.00154 | 0.000666 | -0.00161 |
|  | p=0.985 | p=0.557 | p=0.636 | p=0.309 | p=0.250 | p=0.587 | p=0.480 |
|  | (0.000947) | (0.00128) | (0.00126) | (0.00222) | (0.00132) | (0.00122) | (0.00226) |
| Mean of outcome variable | 34.92 | 34.94 | 34.90 | 34.92 | 34.92 | 34.92 | 34.92 |
| N | 35995 | 16722 | 19273 | 11168 | 24827 | 27352 | 8643 |

SOURCE - Panels of the China Health and Nutrition Survey (CHNS) from 1997 to 2015.

NOTE - The sample is confined to individuals aged 16 and above. The key variable of interest is the daily temperature deviation of the survey date from the weekly average. The weather data is extracted from the closest weather station within 50 miles from the county center. The “abnormal BMI” subsample contains individuals whose BMI≥25 or BMI<18.5 (column (4)), and the “normal BMI” subsample contains individuals with 18.5≤BMI<25 (column (5)). The control variables include individual-level demographic and SES variables, the county-level indices, weather factors other than temperature, the weekly average temperature and other weather factors, individual fixed effects, and time fixed effects. Standard errors are clustered at the county level. * significant at 10%. ** significant at 5%. *** significant at 1%.

*Table S14. The Impact of Daily Deviation from the Weekly Average Temperature on Cognition Proxied by Questionnaire Completeness, using Threshold Regressions*

|  | (1) | (2) | (3) | (4) | (5) | (6) | (7) |
| --- | --- | --- | --- | --- | --- | --- | --- |
| Outcome: Questionnaire completeness of the health and medical service survey | | | | | | | |
| Sample | All | Female | Male | Abnormal BMI | Normal BMI | Rural | Urban |
| $\Delta temp\times(Temperature<77.0℉)$ | 0.000415 | 0.000209 | 0.000570 | 0.000675 | 0.000339 | 0.000778 | -0.000335 |
|  | p=0.579 | p=0.825 | p=0.420 | p=0.527 | p=0.686 | p=0.356 | p=0.719 |
|  | (0.000742) | (0.000940) | (0.000701) | (0.00106) | (0.000834) | (0.000834) | (0.000927) |
| $\Delta temp\times(Temperature\geq77.0℉)$ | -0.00472 | -0.00625 | -0.00341 | -0.00591 | -0.00505 | -0.00592 | 0.0000735 |
|  | p=0.279 | p=0.176 | p=0.438 | p=0.340 | p=0.231 | p=0.267 | p=0.980 |
|  | (0.00432) | (0.00456) | (0.00436) | (0.00614) | (0.00417) | (0.00528) | (0.00299) |
| Mean of outcome variable | 21.97 | 21.97 | 21.97 | 21.97 | 21.97 | 21.97 | 21.97 |
| N | 51063 | 24283 | 26780 | 14481 | 36582 | 37989 | 13074 |

SOURCE - Panels of the China Health and Nutrition Survey (CHNS) from 1989 to 2015.

NOTE - The sample is confined to individuals aged 16 and above. The regime-dependent variable is the daily temperature deviation from the weekly average, and the threshold variable is the daily temperature. The weather data is extracted from the closest weather station within 50 miles from the county center. The “abnormal BMI” subsample contains individuals whose BMI≥25 or BMI<18.5 (column (4)), and the “normal BMI” subsample contains individuals with 18.5≤BMI<25 (column (5)). The control variables include individual-level demographic and SES variables, the county-level indices, weather factors other than temperature, the weekly average temperature and other weather factors, individual fixed effects, and time fixed effects. Standard errors are clustered at the county level. * significant at 10%. ** significant at 5%. *** significant at 1%.

*Table S15. The Impact of Daily Deviation from the Weekly Average Temperature on Cognition Proxied by Questionnaire Completeness, including Questions about Diet and Physical Activities, using Threshold Regressions*

|  | (1) | (2) | (3) | (4) | (5) | (6) | (7) |
| --- | --- | --- | --- | --- | --- | --- | --- |
| Outcome: Questionnaire completeness of the health and medical service survey | | | | | | | |
| Sample | Full | Female | Male | Abnormal BMI | Normal BMI | Rural | Urban |
| $\Delta temp\times(Temperature<77.0℉)$ | 0.000450 | -0.000443 | 0.00117 | 0.00284 | -0.000867 | 0.00130 | -0.00153 |
|  | p=0.690 | p=0.763 | p=0.416 | p=0.243 | p=0.545 | p=0.353 | p=0.519 |
|  | (0.00112) | (0.00146) | (0.00142) | (0.00241) | (0.00142) | (0.00138) | (0.00236) |
| $\Delta temp\times(Temperature\geq77.0℉)$ | -0.00644 | -0.00481 | -0.00756 | -0.00779 | -0.00980 | -0.00741 | -0.00292 |
|  | p=0.371 | p=0.516 | p=0.333 | p=0.372 | p=0.164 | p=0.384 | p=0.480 |
|  | (0.00714) | (0.00735) | (0.00773) | (0.00865) | (0.00694) | (0.00843) | (0.00410) |
| Mean of outcome variable | 34.92 | 34.94 | 34.90 | 34.92 | 34.92 | 34.92 | 34.92 |
| N | 35995 | 16722 | 19273 | 11168 | 24827 | 27352 | 8643 |

SOURCE - Panels of the China Health and Nutrition Survey (CHNS) from 1997 to 2015.

NOTE - The sample is confined to individuals aged 16 and above. The regime-dependent variable is the daily temperature deviation from the weekly average, and the threshold variable is the daily temperature. The weather data is extracted from the closest weather station within 50 miles from the county center. The “abnormal BMI” subsample contains individuals whose BMI≥25 or BMI<18.5 (column (4)), and the “normal BMI” subsample contains individuals with 18.5≤BMI<25 (column (5)). The control variables include individual-level demographic and SES variables, the county-level indices, weather factors other than temperature, the weekly average temperature and other weather factors, individual fixed effects, and time fixed effects. Standard errors are clustered at the county level. * significant at 10%. ** significant at 5%. *** significant at 1%.

*Table S16. The Impact of Daily Deviation from the Weekly Average Temperature on Respondents’ Blood Pressure*

|  | (1) | (2) | (3) | (4) | (5) | (6) |
| --- | --- | --- | --- | --- | --- | --- |
| Outcome: | Systolic blood pressure | | | Diastolic blood pressure | | |
| Weather station (WS) where the  weather data is extracted from | Closest WS  $\leq$50 miles | Weighted weather  data from WSs  $\leq$50 miles | Closest WS  $\leq$30 miles | Closest WS  $\leq$50 miles | Weighted weather  data from WSs  $\leq$50 miles | Closest WS  $\leq$30 miles |
| $\Delta temp$ | -0.0386 | -0.0389 | -0.0280 | -0.0363* | -0.0358 | -0.0151 |
|  | p=0.230 | p=0.237 | p=0.455 | p=0.084 | p=0.122 | p=0.480 |
|  | (0.0317) | (0.0325) | (0.0372) | (0.0206) | (0.0228) | (0.0212) |
| Mean of outcome variable | 118.3 | 118.2 | 118.5 | 77.25 | 77.22 | 77.40 |
| N | 50975 | 50777 | 39495 | 50975 | 50777 | 39495 |

SOURCE - Panels of the China Health and Nutrition Survey (CHNS) from 1989 to 2015.

NOTE - The sample is confined to individuals aged 16 and above. The key variable of interest is the daily temperature deviation of the survey date from the weekly average. The control variables include individual-level demographic and SES variables, the county-level indices, weather factors other than temperature, the weekly average temperature and other weather factors, individual fixed effects, and time fixed effects. Standard errors are clustered at the county level. * significant at 10%. ** significant at 5%. *** significant at 1%.
